# An Anterior Abdominal Abscess as the Initial Presentation of a Perforated Duodenal Ulcer: A Case Report

**DOI:** 10.7759/cureus.44522

**Published:** 2023-09-01

**Authors:** Vishnu R Yanamaladoddi, Akhilesh Gonuguntla, Anila Vasireddy, Nikhil Gopal, Krishna Kalyan Reddy Janumpalli

**Affiliations:** 1 General Surgery, Narayana Medical College & Hospital, Nellore, IND; 2 Department of General Surgery, Kasturba Medical College, Manipal, IND; 3 Department of Gastroenterology, Kasturba Medical College, Manipal, IND; 4 Transitional Medicine, Detroit Medical Center - Sinai Grace Hospital, Detroit, USA

**Keywords:** acute peritonitis, duodenal perforation, enterocutaneous fistula, intestinal perforation, abdominal wall abscess

## Abstract

Duodenal perforation most commonly presents with life-threatening symptoms of acute abdomen. However, in rare cases, a perforation may have an indolent course due to subclinical progression, and the patient may present with complications at the first visit. We present a case of an anterior abdominal abscess as the initial presentation of a duodenal perforation in a 65-year-old female with no pre-morbidities. The patient presented with a painful mass in the right upper quadrant associated with fever. Physical examination revealed a tender, erythematous swelling in the right hypochondrium and lumbar regions with no signs of peritonitis. Contrast-enhanced CT (CECT) of the abdomen showed a subcapsular hepatic abscess with parietal extension, but no signs of hollow viscus perforation were visible. Empirical antibiotics were given, and incision and drainage (I&D) were performed to drain around 100 mL of pus. However, drain on postop day one demonstrated bile suggesting a hollow viscus perforation, which was confirmed by a Gastrografin study.

## Introduction

Duodenal perforation most commonly presents with life-threatening symptoms of acute abdomen. However, in rare cases, a perforation may present with an indolent course when (a) the perforation is very small or opens into an abscess cavity; (b) the patient is using drugs such as an anti-inflammatory medication, painkiller, or recreational substance; or (c) the patient is immunocompromised [1]. Hence, due to the subclinical progression of the perforation, the patient may present with complications at the first visit. Nevertheless, a CT scan would be able to detect the perforation even in the rare instance of a subclinical chronic perforation. An intra-abdominal abscess as the first presentation of a duodenal perforation undetectable on radiological investigations in a patient with no comorbidities presents a rare diagnostic dilemma.

## Case presentation

**Figure 1:**
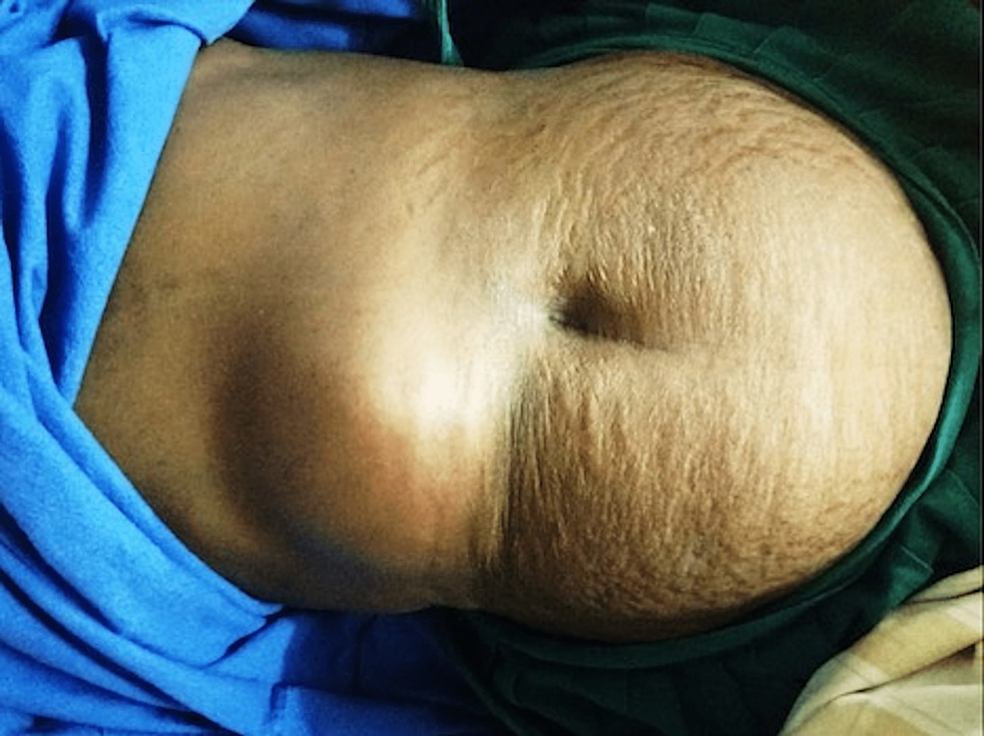
Image showing 15 x 12 cm firm, tender, erythematous swelling in the right hypochondriac and lumbar regions

A 65-year-old female with no pre-morbidities presented with a 20-day history of pain in the abdomen, a 10-day history of abdominal mass, and a 10-day history of intermittent, low-grade fever. The pain in the abdomen was a continuous, dull aching one associated with occasional vomiting. Examination revealed a 15 x 12 cm firm, tender, erythematous swelling in the right hypochondriac and lumbar regions and hepatomegaly. There were no signs of peritonitis (Figure 1). A clinical diagnosis of a liver abscess was made, and the patient was started on an empirical combination of cefoperazone + sulbactam and metronidazole. Investigations revealed leukocytosis with neutrophilia and a negative amoebic serology. Sepsis and tuberculosis were ruled out.

**Figure 2:**
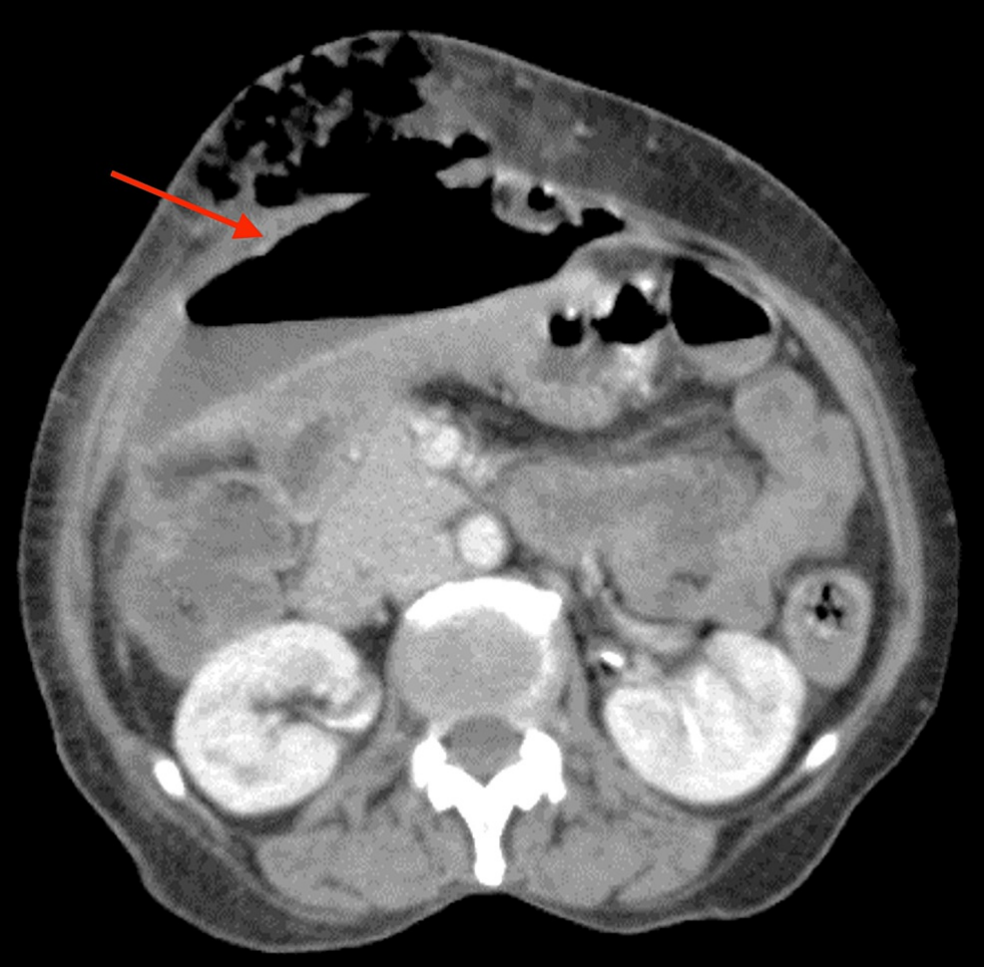
Contrast-enhanced CT showing a collection with enhancing walls The arrow points to air-fluid levels in the costal margin of the right lobe of the liver CT: computed tomography

A contrast-enhanced CT (CECT) of the abdomen and pelvis revealed a collection with enhancing walls and air-fluid levels in the subcapsular region along the costal margin of the right lobe of the liver. A subcutaneous extension through a defect in the right anterior abdominal wall through the rectus abdominis muscle showing signs of subcutaneous emphysema and fat stranding was noted (Figure 2).

**Figure 3:**
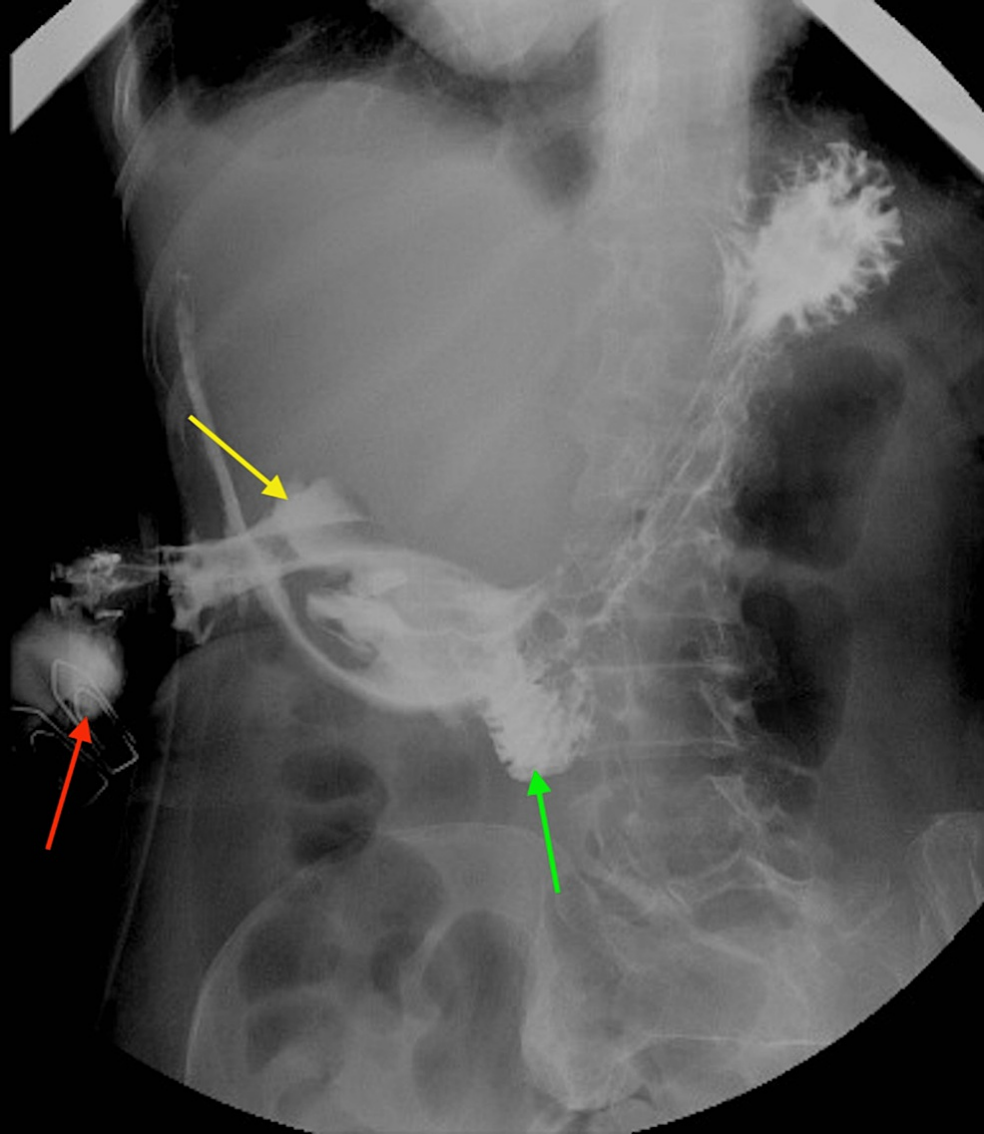
Contrast-enhanced CT - image 2 The red arrow points to the contrast injected into the Foley catheter placed in the abscess cavity. The yellow arrow points to the contrast in the subcapsular abscess cavity. The green arrow points to the contrast in the duodenum CT: computed tomography

The collection was abutting the abdominal wall anterolaterally, the liver posteromedially, and the inferior aspect of the stomach posteriorly. No signs of hollow viscus perforation were identified (Figure 3).

**Figure 4:**
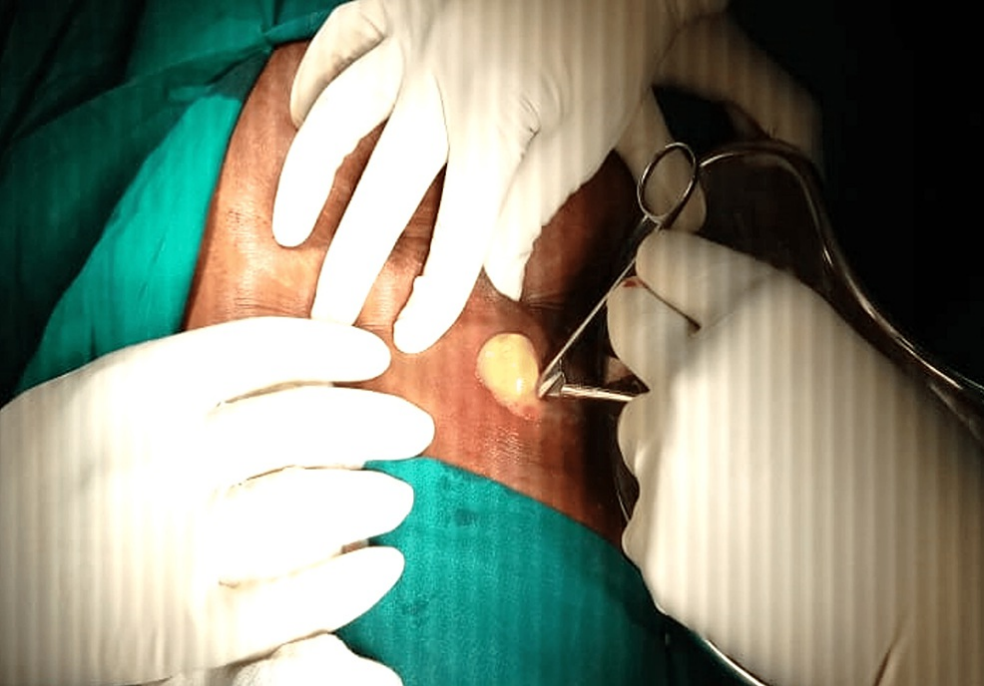
Incision and drainage of pus

This finding led to the final radiological diagnosis of a subcapsular hepatic abscess with parietal extension. However, as the liver parenchyma appeared normal, the presence of an alternative source of the intraabdominal abscess was suspected. Empirical antibiotics were continued, and the patient was planned for incision and drainage (I&D) under local anesthesia. Intraoperatively, I&D was performed by a 2 cm linear transverse incision in the right hypochondrium, draining around 100 mL of pus (Figure 4).

**Figure 5:**
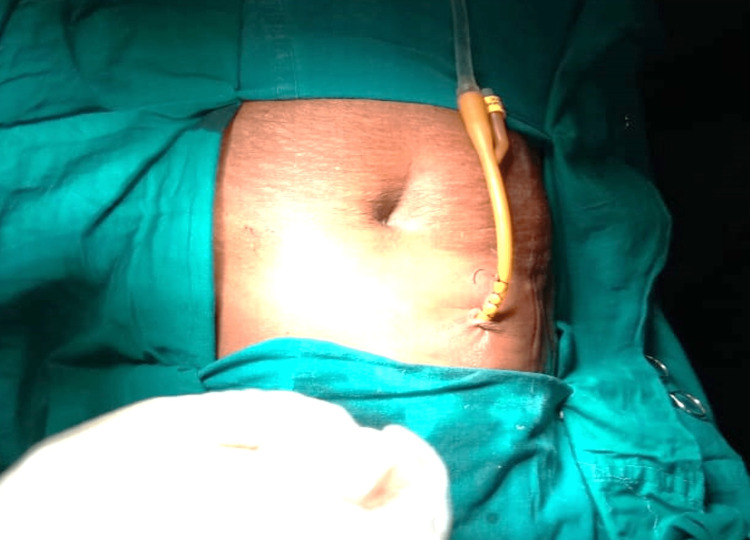
Postoperative drainage with a Foley catheter

In addition, a 2 x 3 cm defect was felt over the rectus sheath communicating with the perihepatic collection. The Foley catheter was inserted for postoperative drainage (Figure 5).

Although the patient improved symptomatically post-surgery, the drain on postop day one showed the presence of bile, suggestive of a hollow viscus perforation with an enterocutaneous fistula (ECF). Hence, conservative management of the ECF was initiated. The high-output fistula drained more than 900 mL of bilious fluid per day over the first five days postoperatively. On postop day six, after drain output had reduced, a Gastrografin study confirmed the presence of a duodenal perforation and warranted the placement of a nasojejunal tube for enteral feeding. 

On postop day eight, the patient developed sepsis with respiratory alkalosis complicated by an acute myocardial infarction, which was confirmed based on elevated procalcitonin, lactate, and troponin I levels. Blood cultures revealed multi-drug-resistant Klebsiella pneumoniae. The patient was immediately started on heparin and scheduled for percutaneous intervention. However, the patient opted to be discharged against medical advice due to affordability issues. She was discharged on a robust medical management course involving cefepime, metronidazole, atorvastatin, ticagrelor, aspirin, metoclopramide, and tramadol, along with a drainage bag and nasojejunal tube in situ. The patient was subsequently lost to follow-up.

## Discussion

Duodenal perforation is the most common cause of peptic ulcer disease-related mortality [2]; 5-10% of all cases of duodenal ulcers perforate with a current mortality rate of <10% with early intervention. However, mortality quickly elevates to 50% with a surgical delay greater than 24 hours [2]. Generally, perforation presents with symptoms of severe, sudden-onset epigastric pain in the setting of a history of peptic ulcer disease in more than two-thirds of patients [2]. The other one-third might exhibit vague symptoms and be identified by the presence of free gas under the diaphragm on a plain abdominal X-ray in more than 90% of cases [2].

An intrabdominal abscess is a rare but significant presentation of subclinical duodenal perforations, as it can be fatal without treatment. A paucity of symptoms, resulting from one or a combination of the following three factors, is the root cause of complications like abdominal sepsis:

(1) The patient may have a very small perforation precluding peritoneal exposure of intestinal contents or may have an ulcer opening into an abscess cavity that may develop due to an incomplete local peritoneal immune response to localize the perforation [1]. Zhonghua et al. have described a case of suspected acute pancreatitis that was later revealed to have been a small duodenal perforation when the patient presented with an anterior abdominal abscess [3]. Aldohuky et al. have reported a case of scrotal abscess secondary to a small posterior duodenal perforation. CT with contrast showed the tracking of oral contrast media along the retroperitoneum into the scrotum [4].

(2) The use of an anti-inflammatory medication, painkiller, or recreational drug may blunt the symptoms of acute abdomen [1]. Shen et al. have reported a case of a duodenal perforation presenting as a subphrenic abscess in a patient on methadone for chronic opioid abuse [1]. Likewise, Mao et al. have described the case of an alcoholic patient with a retroperitoneal abscess secondary to a duodenal perforation [5]. 

(3) The patient may be immunocompromised with impaired host response and perception [2]. Ashfaq et al. have described the case of a patient on immunomodulators and steroids for seronegative rheumatoid arthritis who presented with an intrabdominal abscess in the setting of a subclinical duodenal perforation [6].

In addition, duodenal perforation may present with atypical symptoms in rare cases. Bruner and Gustafson have reported a case of duodenal perforation presenting as acute myocardial infarction that was hypothesized to have developed due to a systemic inflammatory response leading to myocardial dysfunction [7]. Noussios et al. have reported a case of Valentino’s syndrome, which represents a perforated posterior duodenal ulcer presenting as right iliac fossa pain due to tracking of fluid along the retroperitoneum and into the right iliac fossa [8].

Ultrasound is generally the first investigation performed to confirm the suspicion of a liver abscess, as it has a sensitivity of about 85% [9]. However, in our patient, suspected parietal spread necessitated a CECT. Although CECT has a sensitivity of up to 95% and a specificity of up to 100% for perforations, it was likely ineffective in this situation due to potential plugging by omentum or surrounding organs [10]. The abdominal abscess was initially suspected of having originated from a different source of infection when the CT scan revealed a subcapsular liver abscess with parietal extension but normal-looking liver parenchyma.

The treatment of parieto-intraabdominal abscess involves percutaneous I&D according to the Surgical Infection Society (SIS) guidelines [11]. A Foley catheter was placed as the in situ drain and revealed bile on the first postop day, confirming the presence of a hollow viscus perforation. We hypothesize that the perforation formed a localized abscess that communicated with the subcapsular liver space leading to the purulent collection. This collection likely accumulated and burst through the rectus muscle into the subcutaneous space, forming an anterior abdominal abscess with an ECF. Due to the high drain output in our patient, confirmation of the duodenal perforation was delayed until drain output stabilized on postop day six.

## Conclusions

It is important to consider a hollow viscus perforation in the setting of an anterior abdominal abscess as delayed or missed diagnosis can result in unnecessary medical burden on the patient. Although CECT is highly sensitive and specific for detecting hollow viscus perforation, it should be kept in mind that a hollow viscus perforation may not be visible in imaging studies in rare cases, which can lead to a delay in the diagnosis.
